# Frequency of family meals and food consumption in families at high risk of type 2 diabetes: the Feel4Diabetes-study

**DOI:** 10.1007/s00431-022-04445-4

**Published:** 2022-03-30

**Authors:** Lubna Mahmood, Esther M. González-Gil, Peter Schwarz, Sandra Herrmann, Eva Karaglani, Greet Cardon, Flore De Vylder, Ruben Willems, Konstantinos Makrilakis, Stavors Liatis, Violeta Iotova, Kaloyan Tsochev, Tsvetalina Tankova, Imre Rurik, Sándorné Radó, Luis A. Moreno, Yannis Manios, on behalf of the Feel4Diabetes-Study Group, Yannis Manios, Greet Cardon, Jaana Lindström, Peter Schwarz, Konstantinos Makrilakis, Lieven Annemans, Winne Ko, Kalliopi Karatzi, Odysseas Androutsos, George Moschonis, Spyridon Kanellakis, Christina Mavrogianni, Konstantina Tsoutsoulopoulou, Christina Katsarou, Eva Karaglani, Irini Qira, Efstathios Skoufas, Konstantina Maragkopoulou, Antigone Tsiafitsa, Irini Sotiropoulou, Michalis Tsolakos, Effie Argyri, Mary Nikolaou, Eleni-Anna Vampouli, Christina Filippou, Kyriaki Apergi, Amalia Filippou, Gatsiou Katerina, Efstratios Dimitriadis, Tiina Laatikainen, Katja Wikström, Jemina Kivelä, Päivi Valve, Esko Levälahti, Eeva Virtanen, Tiina Pennanen, Seija Olli, Karoliina Nelimarkka, Vicky Van Stappen, Nele Huys, Ruben Willems, Samyah Shadid, Patrick Timpel, Stavros Liatis, George Dafoulas, Christina-Paulina Lambrinou, Angeliki Giannopoulou, Ernest Karuranga, Luis Moreno, Fernando Civeira, Gloria Bueno, Pilar De Miguel-Etayo, Esther Mª Gonzalez-Gil, María L. Miguel-Berges, Natalia Giménez-Legarre, Paloma Flores-Barrantes, Aleli M. Ayala-Marín, Miguel Seral-Cortés, Lucia Baila-Rueda, Ana Cenarro, Estíbaliz Jarauta, Rocío Mateo-Gallego, Violeta Iotova, Tsvetalina Tankova, Natalia Usheva, Kaloyan Tsochev, Nevena Chakarova, Sonya Galcheva, Rumyana Dimova, Yana Bocheva, Zhaneta Radkova, Vanya Marinova, Yuliya Bazdarska, Tanya Stefanova, Imre Rurik, Timea Ungvari, Zoltán Jancsó, Anna Nánási, László Kolozsvári, Csilla Semánova, Éva Bíró, Emese Antal, Sándorné Radó, Remberto Martinez, Marcos Tong

**Affiliations:** 1 Growth, Exercise, Nutrition and Development (GENUD) Research Group, University of Zaragoza, Zaragoza, Spain; 2 Department of Biochemistry and Molecular Biology II, School of Pharmacy, University of Granada, Granada, Spain; 3 Institute of Nutrition and Food Technology “José Mataix”, Center of Biomedical Research, University of Granada. Avda. del Conocimiento S/N, 18016 Armilla, Granada, Spain; 4 Centro de Investigación Biomédica en Red de Fisiopatología de La Obesidad Y Nutrición (CIBERObn), Instituto de Salud Carlos III, Madrid, Spain; 5 Department for Prevention and Care of Diabetes, Medical Faculty Carl Gustav Carus, Technical University of Dresden, Dresden, Germany; 6 Faculty of Medicine, Paul Langerhans Institute Dresden of the Helmholtz Center Munich at University Hospital, TU Dresden, 01307 Dresden, Germany; 7 German Center for Diabetes Research (DZD E.V.), 85764 Neuherberg, Germany; 8 Department of Nutrition and Dietetics, School of Health Science & Education, Harokopio University, Athens, Greece; 9 Department of Movement and Sports Sciences, Ghent University, Ghent, Belgium; 10 Department of Public Health and Primary Care, Ghent University, Ghent, Belgium; 11 National and Kapodistrian University of Athens Medical School, Athens, Greece; 12 Department of Social Medicine and Health Care, Organization Medical University of Varna, Varna, Bulgaria; 13 Department of Diabetology, Clinical Center of Endocrinology, Medical University of Sofia, Sofia, Bulgaria; 14 Faculty of Health Sciences, University of Debrecen, Debrecen, Hungary; 15 Instituto Agroalimentario de Aragón (IA2), Zaragoza, Spain; 16 Instituto de Investigación Sanitaria de Aragón (IIS Aragón), Zaragoza, Spain; 17 Institute of Agri-Food and Life Sciences, Hellenic Mediterranean University Research Centre, Heraklion, Greece

**Keywords:** Family meals, Food consumption, Diet quality, Type 2 diabetes, Parents, Children

## Abstract

A family meal is defined as a meal consumed together by the members of a family or by having ≥ 1 parent present during a meal. The frequency of family meals has been associated with healthier food intake patterns in both children and parents. This study aimed to investigate in families at high risk for developing type 2 diabetes across Europe the association (i) between family meals’ frequency and food consumption and diet quality among parents and (ii) between family meals’ frequency and children’s food consumption. Moreover, the study aimed to elucidate the mediating effect of parental diet quality on the association between family meals’ frequency and children’s food consumption. Food consumption frequency and anthropometric were collected cross-sectionally from a representative sample of 1964 families from the European Feel4Diabetes-study. Regression and mediation analyses were applied by gender of children. Positive and significant associations were found between the frequency of family meals and parental food consumption (*β* = 0.84; 95% CI 0.57, 1.45) and diet quality (*β* = 0.30; 95% CI 0.19, 0.42). For children, more frequent family meals were significantly associated with healthier food consumption (boys, *β* = 0.172, *p* < 0.05; girls, *β* = 0.114, *p* < 0.01). A partial mediation effect of the parental diet quality was shown on the association between the frequency of family meals and the consumption of some selected food items (i.e., milk products and salty snacks) among boys and girls. The strongest mediation effect of parental diet quality was found on the association between the frequency of family breakfast and the consumption of salty snacks and milk and milk products (62.5% and 37.5%, respectively) among girls.

*Conclusions*: The frequency of family meals is positively associated with improved food consumption patterns (i.e., higher intake of fruits and vegetables and reduced consumption of sweets) in both parents and children. However, the association in children is partially mediated by parents’ diet quality. The promotion of consuming meals together in the family could be a potentially effective strategy for interventions aiming to establish and maintain healthy food consumption patterns among children.

*Trial registration*: The Feel4Diabetes-study is registered with the clinical trials registry (NCT02393872), http://clinicaltrials.gov, March 20, 2015.

**Table:** 

**What is Known:** *• Parents’ eating habits and diet quality play an important role in shaping dietary patterns in children* *• Family meals frequency is associated with improved diet quality of children in healthy population*	
**What is New:** *• Frequency of family meals was significantly associated with healthier food consumption among parents and children in families at high risk of type 2 diabetes in six European countries.* *• Parental diet quality mediates the association between family meals frequency and the consumption of some selected food items among children.*

**Supplementary information:**

The online version contains supplementary material available at 10.1007/s00431-022-04445-4.

## Introduction

Children’s optimal nutrition is fundamental for their healthy growth and development [1]. In this sense, the type and variety of food items provided during childhood may influence life-long health and shape long-lasting dietary patterns [2]. In children, unhealthy dietary habits have been associated with adiposity [3] and other comorbidities such as insulin resistance, considered the first stage in the development of type 2 diabetes mellitus (T2DM) among children [3, 4].

It has been found that parents’ eating habits and food preferences play an important role in shaping food intake patterns in children [5] as children consider their parents as role models. Previous studies suggested that parents who are “healthy role models” would be more likely to have children who consume healthier foods [6, 7]. Moreover, parents were found to have a strong influence over the family environment where meals take place and the types and portions of foods provided to children [8].

Family mealtime environment including meal structure, parental modelling, food socialization practices, and types of food provided has great potential to affect the eating behaviors of children [9, 10]. Thus, family meals could offer a promising entry point for change. Although the majority of parents and their children recognize the importance of eating together, they also reported several barriers for sharing mealtimes, including different schedules, implying the difficulty of finding time to share meals [11]. For this reason, “evening meal” or “dinner” is the most common shared meal as families eat together more usually at this meal occasion versus other times of the day [12].

The growing body of scientific evidence has shown that having more frequent family meals is positively associated with improved food intake and enhanced diet quality (DQ) in children [12]. In support of this, children who frequently have shared meals with family show healthier dietary patterns and were found to consume less sodium, sugar, and fat compared to those who seldom do so [13–15]. Cross-sectional and longitudinal studies suggested that regular family meals were associated with higher consumption of fruit and vegetables (FV) in children [16, 17]. Other studies showed that children who eat meals with their family reported healthier dietary intake including higher consumption of fiber, FV, vitamins, and minerals [12]. The high intake of dairy products has also been reported among children who share breakfast with their family [16]. Furthermore, a meta-analysis including over 180,000 children found that children who shared their meals with parents three times or more per week had reduced odds of eating unhealthy food and becoming overweight [15], concluding that family meals’ frequency is positively related to children’s food intake and that further research is needed on how family meals’ quality relates to children’s nutritional intake. Similarly, it has been reported that children who had a lower intake of fried foods and soft drinks frequently consume their meals together with family [13]. Despite the growing interest in this topic, there are still limited studies examining the effects of regular shared family meals and children’s food consumption especially among populations at high risk of developing non-communicable diseases, such as T2DM.

Previous studies associating DQ of parents and children’s intake have shown that improvement in parental DQ was associated with healthier food consumption of children [18, 19]. In addition, more frequent family meals have been positively linked to higher DQ in parents [20] and children [15]. However, no previous study has examined whether parental DQ could have an impact in the association between family meals’ frequency and the food consumption of children. Therefore, the current study attempts to fill gaps in the literature by (1) investigating whether parents who share meals with family have a better food consumption and DQ; (2) examining the association between family meals frequency and children’s consumption of selected food items; (3) assessing the mediation effects of parental DQ on the association between family meals’ frequency and children’s food consumption in families at high risk of T2DM across six European countries.

## Methods

### Study design

Feel4Diabetes-study (Families across Europe following a Healthy Lifestyle for Diabetes prevention) is a cluster randomized study that took place between 2016 and 2018 across six European countries and 11,396 families were included. The study aimed to develop, implement, and evaluate a school- and community-based intervention to promote healthy lifestyle and tackle obesity for the prevention of T2DM among families from vulnerable groups. Thus, an intervention area and a control area in each country were defined. The participating countries were grouped in regions as low-income countries (Bulgaria and Hungary), high-income countries (Belgium and Finland), and countries under austerity measures following the economic crisis (Greece and Spain). Children attending the first three grades of compulsory education in primary schools as well as their parents were invited to participate in the study. Data were collected at baseline (2016), first (2017), and second year (2018) of the program by well-trained researchers. The current paper used the baseline cross-sectional data only. The Feel4Diabetes-study is registered within the clinical trials registry http://clinicaltrials.gov, (NCT02393872). Details of the study protocol have been previously published (https://feel4diabetes-study.eu/) [21].

The “high-risk families” were considered when being at risk for developing T2DM in the following 10 years, predicted by the FINDRISC score, based on T2DM risk estimation, if at least one parent fulfilled the country-specific cut-off point for FINDRISC [17]. FINDRISC is a reliable and valid questionnaire that consisted of eight questions related to age, blood pressure medication, history of high blood glucose, family history of diabetes, body mass index (BMI), waist circumference (WC), physical activity, and consumption of FV [22].

The Feel4Diabetes-study followed the conventions of the Council of Europe Convention on Human Rights and Biomedicine and the Declaration of Helsinki. Ethical approval was provided by the Ethical Committees of all participating European countries including Spain (ethical approval code: CP03/2016), Greece (code: 46/3–4-2015), Finland (code:174/1801/2015), Belgium (code: B670201524237), Bulgaria (code: 52/10–3-201r), and Hungary (code: 20,095/2016/EKU). All parents were informed about the purpose of the study, and they signed a written informed consent for their participation, which gave them the chance to withdraw from the study at any point.

### Study sample

Identified as “high-risk families” were 4484 families at baseline in the Feel4Diabetes-study. In order not to duplicate parental information, since some families included more than one child, one child from each family was randomly included and was linked to the reported parental information. Inclusion criteria were parent with one primary school-aged child having complete data of two questionnaires: food frequency and eating behaviour questionnaires and energy balance–related behaviours questionnaires (one for adult and one for children). From 2648 families that met the inclusion criteria, 702 were excluded for incomplete information and lack of anthropometric measurements, and 1946 were included in this study.

### Food frequency questionnaires

Two self-reported questionnaires, food frequency and eating behaviour questionnaires and energy balance–related behaviour questionnaires, were filled out by one of the parents, who completed these questionnaires both for himself/herself and their child [23]. The initial forms of the Food Frequency Questionnaire (FFQ) were developed in English language and then translated back to the language of each participating country to ensure quality and reliability. Some modifications and additional questions were added which helped to culturally adapt the questionnaire for the target population of the Feel4Diabetes-study across the six countries. The reliability of the validated FFQ was testes in 191 pairs of parents and their children (*N* = 191). Parents completed the questionnaires on two occasions, within a 1–2-week interval. Reliability was tested by the intra-class correlation coefficients (ICC) of test–retest [24].

For the present study, only relevant demographic data and measures on family meals’ frequency, food consumption, and selected food items of children were used.

The frequency of family meals was assessed using the question “how often do your children have the following meals with family including breakfast, lunch and dinner”, which could be answered by choosing one of the following options for each meal occasion: never, less than 1 time/week, 1–2 times/week, 3–4 times/week, 5–6 times/week, and daily. Children’s food consumption was assessed according to specific food groups: milk and milk products, cereals, fat, FV, legumes, red meat, white meat, fish and seafood, nuts, salty snacks, and sweets. The intake of beverages such as water, fruit juices, and soft drinks was also assessed. The answers of food consumption were based on a specified portion size of each food item and included the following: on a weekly (less than 1, 1–2, 3–4, or 5–6 times per week) or daily basis (1–2, 3–4, 5 times, or more per day). The portion size provided for each food item was defined with a household unit and placed under the questions [24]. In this study, the consumption of each food item was converted to daily intake in grams by multiplying the standard portion size by the number of servings consumed.

### Anthropometric measurements

The height and weight of parents were self-reported, while those of children were objectively measured at schools by a well-trained research team. Anthropometric measurements were conducted according to standardized protocols [25] with children standing barefoot in light clothing. Weight was measured by Seca 813 and recorded to the nearest 0.1 kg, and standing height was measured by Seca 217 and recorded to the nearest 0.1 cm. Two readings were obtained out of each measurement and the mean was used for the analysis. BMI was calculated as weight (kg) divided by height (m) squared. Finally, children’s BMI z-scores were calculated according to Cole and Lobstein [26] to obtain an optimal measure for their weight in accordance with their gender and age.

### Measures of parental diet quality

In this study, the parental DQ was assessed using the Healthy Diet Score (HDS), a validated indicator that has been formed based on Feel4Diabetes-study dietary questions and tested before over families at high risk of developing T2DM [27]. The HDS consists of 12 components related to food choices and food behaviours with a special scoring system. The total score ranged from 0 to 100, with higher scores indicating better DQ.

### Parent’s demographic characteristics

Information on demographic characteristics of the parents participating in the Feel4Diabetes-study was collected using self-reported questionnaires in all study participants. The education level of parents was asked in a 5-point Likert-type scale question and responses ranged from “less than 7 years” to “more than 16 years” of education. The questions of marital status included five choices: “single”, “married or cohabiting”, “separated or divorced”, “widowed”, and “other”. Regarding the employment status, parents were asked to identify their main occupation over the last 6 months, and 7 answers were included: “stay at home parent”, “work full-time”, “work part-time”, “unemployed”, “full-time education”, “retired”, and “something else”.

### Statistical analysis

**Fig. 1:**
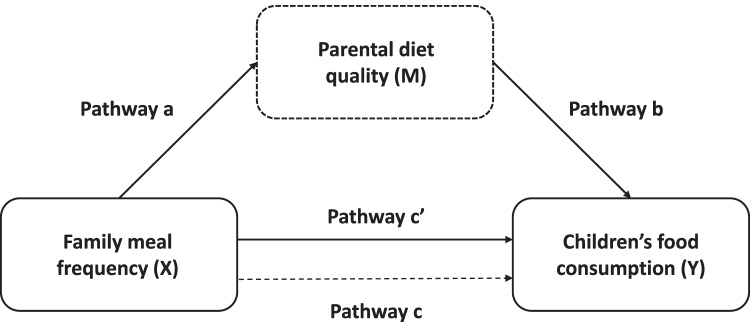
Graphical illustration of the possible interactions between of family meals frequency (X), parental diet quality (M), and children’s food consumption (Y). The mediation analyses adjusted for parent age, gender, country, marital status, education level, and BMI of parents and children. Pathway a: association between (X) and (M). Pathway b: association between (M) and (Y). Pathway c’: direct association between (X) and (Y) after adjustment of (M). (a*b): indirect effect of (M) on the association between (X) and (Y)

Demographic data were described using descriptive measures. Continuous variables were presented using mean and standard deviation, and categorical variables with frequencies and percentages. The normal distribution of variables was checked by Kolmogorov–Smirnov test. The analyses of children were split by gender as new literature on gender differences in eating behaviors among pre-pubertal children identified gender differences in appetitive traits, food intake, food acceptance, self-regulatory eating, and neural response to food images [28]. To assess the relation between family meals’ frequency and food consumption among both parents and children, multiple regression analysis was carried out using the frequency of each family meal as the independent variable and the consumption of selected food items as the dependent variables. The regression analysis was also used to examine the association between family meals’ frequency and parental DQ through using the HDS as the dependent variable. Separate models were run for each meal (i.e., breakfast, lunch, and dinner), and all regression analyses were adjusted for parental age, gender, country, educational level, marital status, socioeconomic status (SES), and BMI of both parents and children, as they were considered as potential predictors of the outcome [12, 16, 18]. Figure 1 provides an outline of the hypothesized relationships between the exposure variable (family meals frequency), potential mediator (parental DQ), and outcome (children’s food consumption). In mediation analysis, the (a-path) represents the association between family meals frequency and the mediator. The (b-path) represents the association between mediator and children’s food consumption used as outcome, adjusted for family meals frequency. The (c’-path) represents the association between family meals frequency and parental DQ and children’s food consumption used as outcome, adjusted for the mediator. The (c-path) represents the total association between family meals’ frequency and the outcome variables. As recommended by Baron and Kenny [29], the following assumptions must be fulfilled to establish a mediation effect: (i) the predictor and outcome variable need to be significantly correlated, and (ii) mediators need to be significantly correlated with both the predictor and outcome variable in order to include them in the model. These assumptions were checked through regression analyses. Covariates included were parent age, gender, country, educational level, marital status, and BMI of parents and children. The indirect effects (a*b) were obtained through conducting Bootstrapping (5000 samples) using the PROCESS macro for SPSS by Preacher and Hayes [30]. Finally, if the effect of (X) on (Y) disappears entirely when (M) is included, complete mediation is indicated. Partial mediation is indicated when the effect of (X) on (Y) is reduced when (M) is included. Significant mediations were then calculated as percentages by dividing (a*b) by the total effect (c-coefficient).

Data were analysed with IBM-SPSS Statistics for Windows (Version 26.0. Armonk, NY: IBM Corp, USA), with a *p* < 0.05 representing statistical significance for all tests.

## Results

### Characteristics of study participants

Descriptive statistics of the sample and variables can be found in Table 1. In total, data of 1946 parents and children from “high-risk” families were analysed (mean age parents, 36.9 ± 4.9 years; 69% mothers; mean age children, 7.1 ± 0.9 years; 52% girls). More than half of the parents were employed (66%) and had a tertiary education of more than 13–14 years (63%).

**Table 1:** Descriptive statistics

**Characteristics**	**Parents**	**Children**
Age (in years)	36.9 (4.9)	7.19 (0.9)
Sex (% female)	69%	52%
Education level (% high education^*^)	63%	−
Employment status (% employed)	66%	−
Marital status (% married)	74%	−
Body weight (kg)	72.8 (16.1)	28.2 (7.3)
Height (cm)	164.6 (7.9)	131.4 (7.5)
^a^BMI (kg/m^2^)	26.4 (5.1)	16.3 (2.8)
BMI z-score		0.68 (1.0)

*N* = 1946 parents and children. This table provides mean (SD) for the continuous variables and frequency (%) for the categorical variables

*13–14 years of education or more

^a^*BMI* body mass index. BMI z-scores were calculated according to Cole et al.

### The frequency of shared family meals for parents and children

The mean of shared family breakfast, lunch, and dinner were 3–4 times, 3–4 times, and 5–6 times a week respectively. Around half of children were sharing breakfast with their parents for more than two times a week, and the majority of children were taking their lunch with one parent at least for more than 3 times weekly. More than 80% of children had more than five times a week family dinner. The percentages of shared family meals for breakfast, lunch, and dinner were almost similar among boys and girls.

### Relationships between the frequency of family meals and parental food consumption

As presented in Table 2, significant associations were found between family meals’ frequency and the food consumption of parents. The shared breakfast was positively associated with an increased intake of milk and milk products, grain bread, and breakfast cereals, fruits, and legumes. The family lunch was positively associated with the consumption of grain groups, FV, and legumes and negatively associated with the intake of sweets. Family dinner was positively related to more consumption of legumes, white meat, and poultry and negatively related to the intake of sweets and sugar-sweetened beverages (SSB).

**Table 2:** Association between family meals’ frequency, parental food consumption, and parental diet quality

	**Breakfast with family**		**Lunch with family**		**Dinner with family**	
	**β (95% CI)**	***p*** **value**	**β (95% CI)**	***p*** **value**	**β (95% CI)**	***p*** **value**
**Parental food consumption (g/day)**						
Milk and milk products ^a^	0.84 (0.57, 1.45)	**0.040**	0.009 (0.01, 0.02)	0.172	0.009 (0.001, 0.05)	0.151
Grain bread and BF cereals ^b^	0.65 (0.27, 1.56)	**0.005**	0.26 (0.09, 0.70)	**0.030**	0.21 (0.09, 0.32)	0.104
Fruits	0.48 (0.34, 1.32)	**0.033**	0.66 (0.21, 2.05)	**0.013**	0.48 (0.28, 0.84)	0.225
Vegetables	0.28 (0.14, 1.24)	0.060	0.39 (0.07, 2.07)	**0.006**	0.08 (0.01, 0.44)	**0.043**
Legumes	0.58 (0.32, 1.06)	**0.010**	0.59 (0.34, 1.00)	**0.045**	0.17 (0.01, 0.20)	0.469
Red meat	0.08 (0.06, 0.17)	0.379	0.08 (0.55, 1.29)	0.463	0.005 (0.001, 0.03)	0.579
White meat and poultry	0.09 (0.03, 0.26)	0.135	0.03 (0.02, 0.08)	0.556	0.67 (0.21, 1.88)	**0.009**
Fish and seafood	0.02 (0.01, 0.03)	0.052	0.05 (0.01, 0.07)	0.278	0.25 (0.08, 0.90)	0.407
Salty snacks	0.06 (0.04, 0.08)	0.070	0.11 (0.85, 0.16)	0.191	0.47 (0.14, 0.97)	0.098
Sweets	0.22 (0.02, 2.53)	0.188	−0.09 (−0.01, −0.17)	**0.012**	−0.13 (−0.05, −0.22)	**0.017**
Sugar sweetened-beverages	0.08 (0.04, 1.63)	0.595	0.65 (0.27, 1.56)	0.059	− 0.09 (−0.03, −0.16)	**0.013**
Parental diet quality (HDS)	0.30 (0.19, 0.42)	**0.001**	0.19 (0.80, 0.26)	**0.038**	−0.15 (0.09, 0.77)	**0.002**

*N* = 1946 parents. Regression analyses were adjusted for parent age, gender, country, marital status, educational level, SES, and BMI of parents and children

*P* < 0·05 (bold indicate significance)

*BF* breakfast, *β* standardized coefficient, *CI* confidence interval

^a^cheese was not counted

^b^rice and pasta were not mentioned under grains group in the questionnaire

### Associations between family meals’ frequency and parental diet quality

Table 2 shows the significant positive associations between parental HDS and the frequency of family meals in all shared meal occasions including breakfast (*β* = 0.30, *p* < 0.001), lunch (*β* = 0.19, *p* < 0.05), and dinner (*β* = 0.15, *p* < 0.01).

### Associations between family meals’ frequency and children’s food consumption

As shown in Table 3, significant associations were found between family meals’ frequency and children’s food consumption of selected food items. More frequent family breakfast was positively associated with the intake of milk and milk products and negatively associated with salty snack consumption among boys and girls. Sharing lunch with family was positively linked to higher intake of fruits and poultry among girls. In boys, frequent family lunch was positively related to increased intake of vegetables. Shared dinner was positively associated with higher intake of vegetables, fish, and seafood among boys. In girls, family dinner was associated with higher consumption of fruits and decreased intake of sweets.

**Table 3:** The associations between family meals’ frequency, parental diet quality, and children’s food consumption

	**Family meals’ frequency** **β (SE)**			**Parental diet quality** **β (SE)**
	**Breakfast**	**Lunch**	**Dinner**	
**Children’s food consumption (g/day)**				
Milk and milk products^a^				
Boys	0.172 (0.04)*	0.096 (0.04)	0.095 (0.02)	0.164 (0.03)*
Girls	0.144 (0.04)**	0.119 (0.02)	0.140 (0.02)	0.182 (0.01)**
Grain bread and BF cereals^b^				
Boys	0.090 (0.02)	0.180 (0.03)	0.100 (0.10)	0.132 (0.01)*
Girls	0.154 (0.09)	0.160 (0.09)	0.097 (0.02)	0.125 (0.06)*
Fruits				
Boys	0.166 (0.05)	0.136 (0.07)	0.149 (0.10)	0.231 (0.02)***
Girls	0.140 (0.05)	0.113 (0.04)**	0.106 (0.05)**	0.103 (0.02)*
Vegetables				
Boys Girls	0.171 (0.08)	0.126 (0.04)*	0.148 (0.06)**	0.109 (0.01)**
Girls	0.079 (0.06)	0.123 (0.01)	0.126 (0.02)	0.271 (0.00)***
Legumes				
Boys	0.084 (0.08)	0.182 (0.08)	0.083 (0.00)	0.070 (0.03)**
Girls	0.106 (0.10)	0.143 (0.09)	0.130 (0.04)	0.052 (0.01)
Red meat				
Boys Girls	0.090 (0.08)	−0.102 (0.00)	0.095 (0.07)	−0.135 (0.00)
Girls	0.102 (0.05)	0.107 (0.09)	-0.093 (0.09)	0.090 (0.01)
White meat and poultry				
Boys	0.079 (0.05)	0.121 (0.02)	0.160 (0.09)	0.036 (0.01)
Girls	0.223 (0.08)	0.092 (0.06)	0.101 (0.01)	0.100 (0.01)*
Salty snacks				
Boys	−0.174 (0.01)**	−0.088 (0.10)	0.120 (0.03)	−0.194 (0.05)*
Girls	−0.136 (0.12)*	0.120 (0.06)	−0.107 (0.05)	−0.282 (0.01)***
Sweets				
Boys	−0.093 (0.09)	0.079 (0.08)	0.095 (0.04)	0.101 (0.03)
Girls	−0.020 (0.05)	0.085 (0.04)	−0.103 (0.09)*	−0.183 (0.02)**
Sugar sweetened-beverages				
Boys	−0.130 (0.00)	0.092 (0.08)	−0.096 (0.05)	−0.104 (0.01)
Girls	−0.129 (0.01)	−0.124 (0.03)	0.102 (0.06)	−0.217 (0.05)**

*N* = 1946 parents and children. Regression analyses were adjusted for parent age, gender, country, marital status, educational level, SES, and BMI of parents and children

*BF* breakfast, *β* standardized coefficient, *SE* standard error

*P < 0·05; **P < 0·01; ***P < 0·001 (indicate significance)

^a^cheese was not counted

^b^rice and pasta were not mentioned under grains group in the questionnaire

### Relationships between parental diet quality and children’s food consumption

Table 3 shows significant associations between parental DQ and children’s food consumption, and this association continued to be observed even after controlling for frequency of family meals. Negative association was found between the HDS of parents and the intake of sweets and SSB among girls only. The DQ of parents was positively associated with the children’s consumption of FV, grains, milk and milk products, and fish and seafood and negatively associated with the intake of salty snacks in both boys and girls.

### Mediation analysis

As presented in Table 4, results indicated a partial mediation effect of the parental DQ on the association between the frequency of family breakfast and boys’ consumption of salty snacks and milk and milk products with 32.7% and 28.4%, respectively. The association between vegetable intake in boys and the family lunch frequency was partially mediated by parental DQ with a percentage of 15.8, and the association between boys’ consumption of vegetables and fish and the shared family dinner was partially mediated by parental DQ with 10.1% and 6.0% respectively. In girls, a partial mediation effect of the mediator was shown on the association between the frequency of family breakfast and consumption of salty snacks, milk and milk products (62.5% vs. 37.5%). The association between the girl’s intake of fruits and poultry with the family lunch frequency was partially mediated with mediator (16.8% vs. 9.3%). The association between the shared family dinner and the intake of fruits and sweets among girl was partially mediated by parental DQ with 13.2% and 24.2%, respectively.

**Table 4:** Mediation analyses for the association between family meals frequency and children’s consumption of selected food items

	Indirect effect (a*b)			**Direct effect (c’- path)**			**Mediation %**		
	**β (95% CI)**			**β (95% CI)**					
**Children’s food consumption (g/day)**	**BF**	**Lunch**	**Dinner**	**BF**	**Lunch**	**Dinner**	**BF**	**Lunch**	**Dinner**
Milk and milk products ^a^									
Boys	0.049 (0.020, 0.063)	**______**	**______**	0.123 (0.089, 0.140)	**______**	**______**	28.40%	**______**	**______**
Girls	0.054 (0.024, 0.080)			0.090 (0.065, 0.103)			37.50%		
Fruits									
Girls	**______**	0.019 (0.008, 0.040)	0.014 (0.007, 0.038)	**______**	0.094 (0.052, 0.157)	0.092 (0.063, 0.019)	**______**	16.80%	13.20%
Vegetables									
Boys	**______**	0.02 (0.009, 0.042)	0.015 (0.008, 0.0403)	**______**	0.16 (0.086, 0.197)	0.133 (0.079, 0.190)	**______**	15.80%	10.10%
White meat and poultry									
Girls	**______**	0.011 (0.007, 0.037)	**______**	**______**	0.107 (0.066, 0.194)	**______**	**______**	9.30%	**______**
Fish and seafood									
Boys	**______**	**______**	0.013 (0.008, 0.026)	**______**	**______**	0.203 (0.109, 0.301)	**______**	**______**	6.00%
Salty snacks									
Boys	−0.057 (−0.084, −0.018)	**______**	**______**	−0.117 (−0.201, −0.096)	**______**	**______**	32.70%	**______**	**______**
Girls	−0.085 (−0.102, −0.045)			−0.051(−0.085, −0.014)			62.50%		
Sweets									
Girls	**______**	**______**	−0.025 (−0.035, −0.014)	**______**	**______**	−0.078 (−0.100, −0.059)	**______**	**______**	24.20%

*N* = 1946 parents and children. Only significant data is presented

*β* standardized coefficient, *CI* confidence intervals. Mediation analyses were adjusted for parent age, gender, country, marital status, educational level, SES, and BMI of parents and children, *BF* breakfast

^a^ cheese was not counted

^b^rice and pasta were not mentioned under grains group in the questionnaire

## Discussion

The main results in the present study suggest that parental food consumption and DQ were significantly associated with the frequency of shared family meals in families at high risk of developing T2DM. Also, a significant association was observed between family meals’ frequency and children’s food consumption. Besides, partial mediation effect of the parental DQ has been identified on the association between the frequency of family meals and the consumption of some food items among boys and girls. All these results were found independently of parent gender, marital status, education level, age, country, SES, and BMI of parents and children.

In this study of a diverse sample of European families, almost 50% of children used to have family breakfast more than five times per week. These findings are consistent with previous research conducted in the USA among children and adolescents, which found similar percentages [12]. In addition, our results indicated that dinner is the most shared meal among children and their parents, most probably because it is the only time of the day when the whole family can get together, especially on working days. This was also supported by previous studies which found that family breakfast occurs less often than family dinners (1.5 breakfast meals versus 4.1 dinner meals per week) [31, 32]. Additionally, a high proportion of school-age children usually consume a school-provided lunch or packed-lunch box at least five times a week [33], and this could explain the reasons behind the low frequency of family lunch.

Positive and significant associations were found in our study between family meals’ frequency and parental DQ. In detail, parents who share their meals with family showed higher scores with better DQ records in all meal occasions including breakfast, lunch, and dinner. These findings help to fill an important gap in the literature as no previous research have examined this association among families at high risk of T2DM and due to the limited research focusing on the DQ of parents related to family meals’ frequency; therefore, a direct comparison could not be made. In addition, in our sample of parents, family meals’ frequency was significantly associated with parents’ food consumption of selected food items. Likewise, cross-sectional and longitudinal studies linking the frequency of family mealtimes with overall dietary intake of parents have also found positive connections [34, 35]. Similar to our results, cross-sectional studies in adults showed that those who share their meals with family more frequently consume less sweets, and SSB, with higher intake of fibres, FV, whole-grain compared to those who do not share their meals with family [36, 37]. Moreover, one systematic review on shared meals with dietary and weight outcomes in youth and adults indicated associations between family meals frequency and improved DQ with increased intake of FV, and decreased intake of soda, fast food, fried foods, higher-fat foods, unhealthy snacks, and cakes [38]. However, the systematic review was based mainly on cross-sectional studies, thus limiting attribution of causality [38].

In our analysis, frequent family meals were associated with a healthier food consumption of children. Children who frequently share breakfast with their families showed increased consumption of milk and milk products and reduced intake of salty snacks. Similarly, a study among American children reported higher consumption of milk and milk products among those who regularly have a family breakfast [31]. However, in contrast with some prior studies [31, 39], we did not find an association between family breakfast and consumption of grain and FV in children. These differences may also be in part related to social desirability bias or less accurate reports of parents [32]. Additionally, in accordance with a previous study in Arab families [40], our results found that more frequent family lunch and dinner was associated with higher intake of FV. Likewise, results of previous studies showed that family lunch and dinner was associated with a higher consumption of FV [31, 40, 41]. The possible explanation of this could be that vegetables are more typically served with lunch and dinner than with breakfast in Europe [38]. Unlike previous research findings [38, 42], in the present study, associations between family meals’ frequency and SSB were not found in all meal occasions including breakfast, lunch, and dinner, which may be the result of low SSB intake in the study population. It is noteworthy that most of the previous studies investigated only one meal occasion and did not specify the meal type, and only few studies examined family breakfast and/or dinner. Besides, very limited studies have examined the effect of shared family lunch on children’s food intake since children tend to consume their lunch at school due to the school schedule in these countries. Moreover, the lack of associations between a specific meal occasion and the intake of some selected food items in our study could be due to culture-specific meal patterns, in which offering this food item may be less dependent upon meal structure than many other foods in the European context. Besides, differences in results in this study compared to that of others could be due to the unadjusted analyses of other studies with uncontrolled confounders or methodological limitations [32].

The present study found that the association between family meals’ frequency and some food items consumed by children occurred, partially, through parental DQ. These results proposed that the children’s consumption of some food items could get affected by the DQ of parents not only by family meals’ frequency. It is noteworthy that this study is the first to examine the mediation effect of parental DQ on the relationship between family meals frequency (i.e., breakfast, lunch, dinner) and children’s food intake. This could, as suggested in previous studies [19, 43] as well as in this study, imply that the DQ of parents play a unique and important role in establishing and maintaining healthy eating behaviours in their children, thereby affecting their food consumption. In this context, our analysis showed significant associations between parental DQ and children’s food consumption among both boys and girls in families at high-risk of T2DM. In detail, a study in children reported lower intake of chocolate, biscuits, cakes, and savoury snacks among children when the parental DQ scores were higher [18]. Similarly, our results found that parental DQ is negatively associated with the consumption of sweets and SSB but only among girls. The possible reason of these results could be that boys tend to consume more sugar than girls in all age-groups [44] and that boys’ food preference and food choice are influenced mainly by taste and not by how healthy foods are, compared to girls [44]. In contrast to our results, a cross-sectional study including primary school children in New Zealand showed no significant associations between DQ of parents and children’s intake of FV [18]. This difference in the results could be due to the use of a different tool to assess the parental DQ compared to the tool used in this study (i.e., Diet Quality Index (DQI) vs. HDS).

This study has some limitations. Firstly, self-reported questionnaires were used for collecting the food consumption data, a method which is not able to adequately determine absolute food intakes compared to other methods (i.e., 24-h dietary recall). Secondly, the cross-sectional nature of this study did not provide information in determining the cause-and-effect association. Another limitation of this study is that children’s data were based on parental report, and therefore, a bias must be considered. However, there are several strengths in this study that need to be mentioned. To the best of our knowledge, the present study is the first to examine the association between family meals’ frequency and parental DQ and parent’s and children’s food consumption in European families at high risk of developing T2DM. This study also provides a unique opportunity in measuring the effect of each type of family meals (i.e., breakfast, lunch, and dinner) on parent’s and children’s food intake. Besides, the anthropometric measurements were obtained by well-trained researchers using highly validated and standardized procedures to ensure and increase accuracy. In addition, the sample size had been selected from a wide geographical spread, including six European countries with a large cultural and dietary diversity, which increases the generalizability of the results.

## Conclusion

Family meals’ frequency was associated with parental DQ and food consumption, while there was also an association between family meals frequency and food consumption of children in families with an increased risk of developing T2DM. Besides, parental DQ partially mediates the association between family meals frequency and children’s consumption of some selected food items. Parents are usually considered as role models, who set the rules for their children’s food intake and dietary habits, while family mealtime environment could have a great potential to change the eating behaviors of children. Therefore, improving parental DQ and increasing the frequency of family meals, both factors could have an impact in children’s food consumption and offer a promising entry point for change through limiting the unhealthy eating habits of children and thereby prevent childhood obesity and T2DM. Further studies are needed to examine the prolonged effects of family meals’ frequency and parental DQ on food consumption of children in families at high risk of T2DM.

## Supplementary information

Below is the link to the electronic supplementary material.

Supplementary file1 (DOCX 14 KB)

## Data Availability

The data of the present study are available for further scientific analysis from the corresponding author on reasonable request.

## Abbreviations

BMI: Body mass index
DQ: Diet quality
DQI: Diet Quality Index
Feel4Diabetes: Families across Europe following a Healthy Lifestyle for Diabetes Prevention
FFQ: Food Frequency Questionnaire
FINDRISC: Finnish Diabetes Risk Score
FV: Fruit and vegetables
HDS: Healthy Diet Score
SES: Socioeconomic status
SSB: Sugar-sweetened beverages
T2DM: Type 2 diabetes mellitus
WC: Waist circumference

## References

[1] Golley RK, Smithers LG, Mittinty MN, Emmett P, Northstone K, Lynch JW (2013). Diet quality of U.K. infants is associated with dietary, adiposity, cardiovascular, and cognitive outcomes measured at 7–8 years of age. J Nutr.

[2] Skinner JD, Carruth BR, Wendy B, Ziegler PJ (2002). Children’s food preferences: a longitudinal analysis. J Am Diet Assoc.

[3] Roblin L (2007). Childhood obesity: food, nutrient, and eating-habit trends and influences. Appl Physiol Nutr Metab.

[4] Annalisa B, Simone F, Laura C, Giovanni P, Francesco C (2016). Role of nutrition in preventing insulin resistance in children. J Pediatr Endocrinol Metab.

[5] Tang D, Bu T, Dong X (2020). Are parental dietary patterns associated with children’s overweight and obesity in China. BMC Pediatr.

[6] Mahmood L, Flores-Barrantes P, Moreno LA, Manios Y, Gonzalez-Gil EM (2021). The influence of parental dietary behaviors and practices on children’s eating habits. Nutrients.

[7] Benton D (2004). Role of parents in the determination of the food preferences of children and the development of obesity. Int J Obes Relat Metab Disord.

[8] Coto J, Pulgaron ER, Graziano PA (2019). Parents as role models: associations between parent and young children’s weight, dietary intake, and physical activity in a minority sample. Matern Child Health J.

[9] Ek Litterbach, Campbell K, Spence A (2017). Family meals with young children: an online study of family mealtime characteristics, among Australian families with children aged six months to six years. BMC Public Health.

[10] Haines J, Haycraft E, Lytle L, Nicklaus S, Kok FJ, Merdji M, Fisberg M, Moreno LA, Goulet O, Hughes SO (2019). Nurturing children’s healthy eating: position statement. Appetite.

[11] Boutelle K, Birnbaum A, Lytle L, Murray D, Story M (2003). Associations between perceived family meal environment and parent intake of fruit, vegetables, and fat. J Nutr Educ Behav.

[12] Gillman MW, Rifas-Shiman SL, Frazier AL, Rockett HR, Camargo CA, Field AE, Berkey CS, Colditz GA (2000). Family dinner and diet quality among older children and adolescents. Arch Fam Med.

[13] Kalish N (2001). Why family dinner is worth it. Parenting Mag.

[14] Sami W, Ansari T, Butt NS, Hamid MRA (2017). Effect of diet on type 2 diabetes mellitus: a review. Int J Health Sci (Qassim).

[15] Dallacker M, Hertwig R, Mata J (2018). The frequency of family meals and nutritional health in children: A meta-analysis. Obes Rev.

[16] Caldwell AR, Terhorst L, Skidmore ER, Bendixen RM (2018). Is frequency of family meals associated with fruit and vegetable intake among preschoolers?. A logistic regression analysis. J Hum Nutr Diet.

[17] Pearson N, Biddle SJ, Gorely T (2009). Family correlates of breakfast consumption among children and adolescents. A systematic review. Appetite.

[18] Davison B, Saeedi P, Black K, Harrex H, Haszard J, Meredith-Jones K, Quigg R, Skeaff S, Stoner L, Wong JE, Skidmore P (2017). The association between parent diet quality and child dietary patterns in nine- to eleven-year-old children from Dunedin. New Zealand Nutrients.

[19] Flórez KR, Richardson AS, Ghosh-Dastidar MB, Beckman R, Huang C, Wagner L, Dubowitz T (2017). Improved parental dietary quality is associated with children’s dietary intake through the home environment. Obes Sci Pract.

[20] Welsh M, French A, Wall M (2011). Examining the relationship between family meal frequency and individual dietary intake: does family cohesion play a role?.. J Nutr Educ Behav.

[21] Manios Y, Androutsos O, Lambrinou CP, Cardon G, Lindstrom J, Annemans L, Mateo-Gallego R, de Sabata MS, Iotova V, Kivela J, Martinez R, Moreno LA, Rurik I, Schwarz P, Tankova T, Liatis S, Makrilakis K (2018). A school- and community-based intervention to promote healthy lifestyle and prevent type 2 diabetes in vulnerable families across Europe: design and implementation of the Feel4Diabetes-study. Public Health Nutr.

[22] Makrilakis K, Liatis S, Grammatikou S, Perrea D, Stathi C, Tsiligros P, Katsilambros N (2011). Validation of the Finnish diabetes risk score (FINDRISC) questionnaire for screening for undiagnosed type 2 diabetes, dysglycaemia and the metabolic syndrome in Greece. Diabetes Metab.

[23] Latomme J, Van Stappen V, Cardon G, Morgan PJ, Lateva M, Chakarova N, Kivelä J, Lindström J, Androutsos O, González-Gil EM, De Miguel-Etayo P, Nánási A, Kolozsvári LR, Manios Y, De Craemer M (2018). The association between children’s and parents’ co-TV viewing and their total screen time in six European countries: cross-sectional data from the Feel4diabetes-Study. Int J Environ Res Public Health.

[24] Anastasiou CA, Fappa E, Zachari K, Mavrogianni C, Van Stappen V, Kivelä J, Virtanen E, González-Gil EM, Flores-Barrantes P, Nánási A, Semánová C, Dimova R, Usheva N, Iotova V, Cardon G, Manios Y, Makrilakis K, Feel4Diabetes-study group (2020). Development and reliability of questionnaires for the assessment of diet and physical activity behaviors in a multi-country sample in Europe the Feel4Diabetes Study. BMC Endocr Disord.

[25] Androutsos O, Anastasiou C, Lambrinou C, Mavrogianni C, Cardon G, Van Stappen V, Kivelä J, Wikström K, Moreno L, Iotova V, Tsochev K, Chakarova N, Ungvári T, Jancso Z, Makrilakis K, Manios Y (2020). Intra- and inter- observer reliability of anthropometric measurements and blood pressure in primary schoolchildren and adults: the Feel4Diabetes-study. BMC Endocr Disord.

[26] Cole TJ, Lobstein T (2012). Extended international (IOTF) body mass index cut-offs for thinness, overweight and obesity. Pediatr Obes.

[27] Virtanen E, Kivelä J, Wikström K, Lambrinou CP, De Miguel-Etayo P, Huys N, Vraukó-Tóth K, Moreno LA, Usheva N, Chakarova N, Rado SA, Iotova V, Makrilakis K, Cardon G, Liatis S, Manios Y, Lindström J, Feel4Diabetes research group (2020). Feel4Diabetes healthy diet score: development and evaluation of clinical validity. BMC Endocr Disord.

[28] Keller K, Kling SMR, Fuchs B, Pearce AL, Reigh NA, Masterson T, Hickok K (2019). A biopsychosocial model of sex differences in children’s eating behaviors. Nutrients.

[29] Baron RM, Kenny DA (1986). The moderator-mediator variable distinction in social psychological research: conceptual, strategic, and statistical considerations. J Pers Soc Psychol.

[30] Preacher KJ, Hayes AF (2008). Asymptotic and resampling strategies for assessing and comparing indirect effects in multiple mediator models. Behav Res Methods.

[31] Larson N, MacLehose R, Fulkerson JA, Berge JM, Story M, Neumark-Sztainer D (2013). Eating breakfast and dinner together as a family: associations with sociodemographic characteristics and implications for diet quality and weight status. J Acad Nutr Diet.

[32] Horning ML, Fulkerson JA, Friend SE, Neumark-Sztainer D (2016). Associations among nine family dinner frequency measures and child weight, dietary, and psychosocial outcomes. J Acad Nutr Diet.

[33] Morrison M (1996). Sharing food at home and school: perspectives on commensality. The Sociological Review.

[34] Larson NI, Perry CL, Story M, Neumark-Sztainer D (2006). Food preparation by young adults is associated with better diet. Quality J Am Diet Assoc.

[35] Larson NI, Neumark-Sztainer D, Hannan PJ, Story M (2007). Family meals during adolescence are associated with higher diet quality and healthful meal patterns during young adulthood. J Am Diet Assoc.

[36] O’Dwyer NA, Gibney MJ, Burke SJ, McCarthy SN (2005). The influence of eating location on nutrient intakes in Irish adults: Implications for developing food-based dietary guidelines. Public Health Nutr.

[37] Naska A, Katsoulis M, Orfanos P, Lachat C, Gedrich K, Rodrigues SS, Freisling H, Kolsteren P, Engeset D, Lopes C, Elmadfa I, Wendt A, Knüppel S, Turrini A, Tumino R, Ocké MC, Sekula W, Nilsson LM, Key T, Trichopoulou A, HECTOR Consortium (2015). Eating out is different from eating at home among individuals who occasionally eat out. A cross-sectional study among middle-aged adults from eleven European countries. Br J Nutr.

[38] Fulkerson JA, Larson N, Horning M, Neumark-Sztainer D (2014). A review of associations between family or shared meal frequency and dietary and weight status outcomes across the lifespan. J Nutr Educ Behav.

[39] Andaya A, Arredondo E, Alcaraz J, Lindsay P, Elder J (2011). The association between family meals, TV viewing during meals, and fruit, vegetables, soda, and chips intake among Latino children. J Nutr Educ Behav.

[40] Alamri E (2020). Family meal associated with better dietary quality during adolescence. Med Sci.

[41] Suggs S, Bella S, Rangelov N, Vidal P (2018). Is it better at home with my family? The effects of people and place on children's eating behavior. Appetite.

[42] Hillesund ER, Sagedal LR, Bere E, Øverby NC (2021). Family meal participation is associated with dietary intake among 12-month-olds in Southern Norway. BMC Pediatr.

[43] Robson SM, Couch SC, Peugh JL, Glanz K, Zhou C, Sallis JF, Saelens BE (2016). Parent diet quality and energy intake are related to child diet quality and energy intake. J Acad Nutr Diet.

[44] Ervin RB, Kit BK, Carroll MD, Ogden CL (2012). Consumption of added sugar among U.S. children and adolescents, 2005–2008. NCHS Data Brief.

